# Supplementary material for: Religious beliefs and practices, political orientation, and distrust in healthcare predict attitudes toward mRNA vaccines in the United States
Source: PLOS Glob Public Health. 2026 Sep 9;6(9):e0006330. doi: 10.1371/journal.pgph.0006330 (PMC13557370; doi:10.1371/journal.pgph.0006330)
Supplement: Supplementary file 1 [file pgph.0006330.s001.pdf]

Supplemental Material

Table of Contents

Section	Page Number
Steps for Evaluating Reliability of Model from Backward Chunkwise Elimination Procedure	2
Backward Chunkwise Elimination Model Building Procedure	3
Assumption Checking for Analysis of Covariance (ANCOVA) Models	4
Assumption Checking for Regression Model Predicting Support and Concerns about mRNA vaccines	5
Supplemental Table 1. Maximum Model for Predicting Support for mRNA vaccines	6-7
Supplemental Table 2. Maximum Model for Predicting Concerns about mRNA vaccines	8-9
Supplemental References	10

Steps for Evaluating Reliability of Model from Backward Chunkwise Elimination Procedure[1]

1. After conducting the backward chunkwise elimination procedure with the training dataset, we record the R^2 vector of parameter estimates from the finalized model.
2. Next, we compute $\hat{Y}_{2|1} = X_2\hat{\beta}_1$, where X_2 is the holdout dataset. Essentially, we used the estimated prediction equation from the training group to compute predicted values of the outcome in the holdout sample.
3. Next, we compute the squared cross-validation correlation: $R_*^2(2) = r^2(Y_2, \hat{Y}_{2|1})$
4. Next, we compute the shrinkage on cross-validation: $R^2(1) - R_*^2(2)$
5. While there are no firm rules as to how large a shrinkage must be to cast doubt on reliability, Kleinbaum et al.[1] suggests that a fitted model is unreliable is the shrinkage is .90 or more, while values less than .10 indicate a reliable model.

Backward Chunkwise Elimination Model Building Procedure[1]

Two backward chunkwise elimination model building procedures [1] were conducted to predict support and concerns in the training group sample. The elimination procedure was first applied to "chunks" of variables (i.e., variables grouped together for conceptual similarity), then applied to individual variables that remain in "chunks" that survived the initial round of elimination. For each round of elimination, the model building procedure consisted of five steps.

1. Specify the full model as the base, with $p = k$ predictors, where k equals to the full number of chunks/variables in the model
2. Fit all p-1 variable models, defined by deleting one chunk/variable from the base model
3. For each model, compute the added-last test for the candidate chunk/variable
4. Find the minimum test statistics, $F_{p.}$
    - If statistically significant (i.e., $F_p > F_{CRIT}$, where α is based on a Sidak-Bonferroni corrected test)[2], stop and choose model p
    - If not statistically significant (i.e., $F_p < F_{CRIT}$), delete the chunk/predictor with the minimum test statistic and reduce p by 1.
5. Go back to Step 2 and repeat the process

Once a final model was determined, we then evaluated the generalizability of the model by evaluating the previously mentioned shrinkage on cross validation value.

Assumption Checking for Analysis of Covariance (ANCOVA) Models Predicting Support and Concerns about mRNA Vaccines

For the ANCOVA models predicting support and concerns about mRNA vaccines, examination of residual and normal probability plots did not suggest gross violations pertaining to linearity, normality, and homoscedasticity assumptions. Examination of interaction terms between each covariate and religious and non-religious groups suggested very small to small effect sizes (partial η^2s $\leq .028$)[3], suggesting no gross violation pertaining to homogeneity of regression slopes for the ANCOVA.

Assumption Checking for Regression Models Predicting Support and Concerns about mRNA Vaccines

For the backward chunkwise elimination procedure predicting support and concerns about mRNA vaccines, we only examined regression assumptions for the maximum models in the training sample and final models in the holdout sample.[1] Examination of residual and normal probability plots for the maximum and final models for both outcomes did not suggest gross violations pertaining to linearity, normality, and homoscedasticity assumptions. Tolerance, variance inflation factor, and condition indices values suggested no significant multicollinearity concerns among the predictors in the maximum and final models.

Supplemental Table 1. Maximum model for predicting support for mRNA vaccines

No.	Model/Predictor	*F*	*df*	*p*	Adjusted $R^2/\Delta R^2$
	Maximum Model				.34
1	Genetic Knowledge Index	5.87	1	.02	0.00
2	Distrust towards the health care system	58.81	1	<.001	0.02
3	Integration of religious or spiritual beliefs in daily living	0.09	1	.77	0.00
4	Fundamentalist religious beliefs	8.55	1	.00	0.00
5	Acceptance of evolution	34.42	1	<.001	0.01
6	Beliefs about God in the body	2.54	1	.11	0.00
7	Closet discrimination: felt need to conceal religious identity from others; Belief that others discriminate against them for their religion	3.97	2	.02	0.00
8	Belief that God controls everything	0.26	1	.61	0.00
9	Spiritual community's support for liberal reproductive & end of life views; Spiritual community's support for community health	64.93	2	<.001	0.05
10	Private prayer frequency; Private prayer time	1.10	2	.34	0.00
11	Meditation frequency; Meditation time	3.50	2	.03	0.00
12	Frequency of volunteering; Attendance frequency in religious or spiritual group activities	6.14	2	<.001	0.00
13	Age	0.33	1	.57	0.00
14	Health in the last four weeks	0.08	1	.78	0.00
15	Education level	5.15	1	.02	0.00
16	Household income	8.13	1	.00	0.00
17	Political orientation	72.54	1	<.001	0.03
18	Suburban[a], urban, or rural status	0.91	2	.41	0.00
19	Catholic[a], Jewish, Muslim, Spiritual, EvangelicalProtestant, MainlineProtestant, BlackProtestant	1.29	6	.26	0.00
20	Female[a], male, other	0.20	2	.82	0.00

21	Non-Hispanic/Latino[a], HispanicLatino, Prefer not to answer	0.92	2	.40	0.00
22	White[a], Native American, Asian, Black, Pacific Islander	1.26	4	.28	0.00

The full set of 27 predictors are grouped into 22 groups to accommodate dummy variables and reduce the overall number of predictors in the model.

[a]Reference categories

Supplemental Table 2. Maximum model for predicting concerns about mRNA vaccines

No.	Model/Predictor	*F*	*df*	*p*	Adjusted $R^2/\Delta R^2$
	Maximum Model				.37
1	Genetic Knowledge Index	4.01	1	.05	0.00
2	Distrust towards the health care system	159.09	1	<.001	0.06
3	Integration of religious or spiritual beliefs in daily living	0.75	1	.39	0.00
4	Fundamentalist religious beliefs	8.59	1	.00	0.00
5	Acceptance of evolution	7.13	1	.01	0.00
6	Beliefs about God in the body	15.43	1	<.001	0.01
7	Religious discrimination: Felt need to conceal religious identity from others; Belief that others discriminate against them for their religion	4.74	2	.01	0.00
8	Belief that God controls everything	3.73	1	.05	0.00
9	Spiritual community's support for liberal reproductive & end of life views; Spiritual community's support for community health	24.37	2	<.001	0.02
10	Private prayer frequency; Private prayer time	0.83	2	.44	0.00
11	Meditation frequency; Meditation time	2.11	2	.12	0.00
12	Frequency volunteer; Attendance frequency in religious or spiritual group activities	3.44	2	.03	0.00
13	Age	0.31	1	.58	0.00
14	Health in the last four weeks	0.15	1	.70	0.00
15	Education level	4.70	1	.03	0.00
16	Household income	1.95	1	.16	0.00
17	Political orientation	87.37	1	<.001	0.03
18	Suburban[a], urban, or rural status	0.20	2	.82	0.00
19	Catholic[a], Jewish, Muslim, Spiritual, EvangelicalProtestant, MainlineProtestant, BlackProtestant	1.73	6	.11	0.00
20	Female[a], male, other	0.95	2	.39	0.00

21	Non-Hispanic/Latino[a], HispanicLatino, Prefer not to answer	0.12	2	.89	0.00
22	White[a], Native American, Asian, Black, Pacific Islander	0.25	4	.91	0.00

The full set of 27 predictors are grouped into 22 groups to accommodate dummy variables and reduce the overall number of predictors in the model.

[a]Reference categories

References

1. Kleinbaum DG, Kupper LL, Nizam A, Muller KE. Applied Regression Analysis and Other Multivariable Methods. 4th ed: Duxbury Press; 2008.
2. Šidák Z. Rectangular Confidence Regions for the Means of Multivariate Normal Distributions. Journal of the American Statistical Association. 1967;62(318):626-33. doi: 10.1080/01621459.1967.10482935.
3. Cohen J. A power primer. Psychological Bulletin. 1992;112(1):155-9. doi: 10.1037//0033-2909.112.1.155.
